# Sex differences in leptin levels but not in the leptin response to sacubitril/valsartan in patients with heart failure with reduced ejection fraction

**DOI:** 10.3389/fcvm.2026.1922853

**Published:** 2026-09-10

**Authors:** Zornitsa Shomanova, Bernhard Ohnewein, Dilvin Semo, Vera Paar, Tobias Fröhling, Jürgen R. Sindermann, Uta C. Hoppe, Michael Lichtenauer, Maciej Zechowicz, Lukas J. Motloch, Rudin Pistulli

**Affiliations:** 1 Interdisciplinary Heart Failure Section, University Hospital Münster, Münster, Germany; 2 Department of Internal Medicine II, Paracelsus Medical University, Salzburg, Austria; 3 Department of Cardiology I, Coronary and Peripheral Vascular Disease, Heart Failure, University Hospital Münster, Münster, Germany; 4 Department of Cardiology and Internal Medicine, School of Medicine, Collegium Medicum, University of Warmia and Mazury, Olsztyn, Poland; 5 Department of Cardiology, Kepler University Hospital, Johannes Kepler University, Linz, Austria; 6 Department of Internal Medicine II, Salzkammergut Klinikum, OÖG, Vöcklabruck, Austria; 7 Medical Clinic I, Marienhospital Papenburg Aschendorf GGmbH, Papenburg, Germany

**Keywords:** biomarkers, heart failure, HFrEF, leptin, sacubitril/valsartan, sex differences

## Abstract

**Background:**

Leptin is an adipokine involved in metabolic regulation and neurohormonal activation and has been implicated in the pathophysiology of heart failure. Sacubitril/valsartan (angiotensin receptor–neprilysin inhibitor, ARNI) improves outcomes in patients with heart failure with reduced ejection fraction (HFrEF) and may also influence metabolic pathways. However, it remains unclear whether ARNI therapy affects circulating leptin levels differently in men and women.

**Methods:**

In this observational study, patients with HFrEF from two clinical centres were evaluated before and after initiation of sacubitril/valsartan therapy. Serum leptin levels, N-terminal pro-B-type Natriuretic Peptide (NT-proBNP), echocardiographic parameters and New York Heart Association (NYHA) class were assessed at baseline and follow-up. Sex-specific differences in leptin levels and leptin dynamics were analysed.

**Results:**

A total of 74 patients (20 women and 54 men) were included. Women exhibited significantly higher leptin levels compared with men at baseline (39.78 ± 31.95 vs. 10.39 ± 17.04 ng/mL, *p* < 0.001) despite similar body mass index (BMI) values (ns). Following initiation of ARNI therapy, leptin levels increased significantly in both women (39.78 ± 31.95 to 50.10 ± 41.28, *p* = 0.041) and men (10.39 ± 17.04 to 12.49 ± 16.62, *p* = 0.006). However, the magnitude of absolute and relative leptin change did not differ significantly between sexes. Improvements in left ventricular ejection fraction (LVEF) were observed in both groups, while changes in NT-proBNP and NYHA class showed different within-group patterns but did not differ significantly between sexes.

**Conclusion:**

Women with HFrEF showed markedly higher circulating leptin levels than men at both baseline and follow-up. Leptin levels increased significantly in both sexes. However, there were no significant differences in absolute or relative leptin changes between women and men. Sex influences baseline leptin concentrations, but not the response to ARNI therapy. Further prospective studies are needed to clarify the mechanisms and clinical significance of leptin changes during sacubitril/valsartan treatment.

## Introduction

A multitude of metabolic and neurohormonal mechanisms have been identified as playing a significant role in the pathophysiology of heart failure with reduced ejection fraction (HFrEF). Reduced cardiac output in heart failure activates compensatory neurohormonal pathways, particularly the sympathetic nervous system and the renin–angiotensin–aldosterone system (RAAS). While these compensatory mechanisms initially maintain cardiovascular stability, chronic activation contributes to adverse cardiac remodeling and progression of heart failure (1). This mechanism is among the most significant in disease progression and represent a primary therapeutic target (1).

Angiotensin receptor–neprilysin inhibitors (ARNI) are a cornerstone of contemporary pharmacological therapy for heart failure. Sacubitril/valsartan has been demonstrated to block the RAAS system and simultaneously inhibit neprilysin, thereby increasing the concentration of natriuretic peptides. This causes increased diuresis, vasodilation and a reduction in preload and afterload, which improves haemodynamic function (2). The PARADIGM-HF study demonstrated that these effects result in a significant reduction in cardiovascular death, hospitalization for heart failure, and cardiovascular and overall mortality (2–4).

Sacubitril/valsartan exerts its effects not only at the neurohormonal but also at the metabolic level. Several studies demonstrate that the medication combination of sacubitril/valsartan enhance peripheral insulin sensitivity in patients who are obese and hypertensive (5, 6). Because leptin plays an important role in insulin sensitivity and lipid metabolism, it may represent a potential link between the metabolic effects of sacubitril/valsartan and adipose tissue function (7).

The role of leptin in heart failure remains incompletely understood. Alterations in leptin signaling can impair cardiac function (8). Experimental studies have shown that impaired leptin signalling in leptin-deficient animal models is associated with cardiac dysfunction and adverse remodelling, whereas leptin replacement may improve cardiac function and outcomes (9). This finding suggests that leptin may play a protective cardiac role.

Whether this protective signal also holds in the clinical setting of heart failure, however, is far less certain. However, the results of clinical studies have been inconsistent. The hormone leptin influences neurohumoral activity (10–12) and thus stimulates the production of aldosterone (13, 14). Elevated levels of circulating leptin have been observed in patients diagnosed with heart failure. Leptin has also been linked to clinical prognosis (15–21). Furthermore, leptin secretion appears to differ between men and women, with potentially distinct physiological effects (22, 23). Taken together, these findings leave unresolved whether elevated leptin in heart failure reflects a compensatory, protective response or contributes to disease progression.

Sex-specific aspects of cardiovascular pharmacology have gained increasing attention, particularly with regard to differences in drug pharmacokinetics between women and men. Differences in body composition, enzyme activity, and hormone status could lead to different effects and side effects (24, 25). Therefore, the response to ARNI therapy in patients with HFrEF may vary according to gender. However, it remains unclear whether ARNI therapy influences circulating leptin levels differently in men and women and whether leptin dynamics are associated with clinical or hemodynamic changes in heart failure.

The present study aims to investigate whether sex-specific differences exist in leptin levels and leptin responses following initiation of sacubitril/valsartan therapy in patients with HFrEF.

## Methods

The current observational study included patients from two university hospitals: Münster University Hospital (Germany) and Salzburg University Hospital (Austria). The study population was recruited from two Central European tertiary care centres and consisted predominantly of patients of Caucasian origin. From March 2019 to December 2020, a total of 74 patients (20 women and 54 men) with reduced left ventricular (LV) function of ischemic and non-ischemic origin were examined. The patients presented for routine checkups or with progression of chronic heart failure and corresponding symptoms, such as dyspnea or worsening New York Heart Association (NYHA) class. The patients were predominantly classified as NYHA classes II to III. Body mass index (BMI) did not differ statistically between sexes, although it tended to be higher in women (BMI women 30.87 ± 6.84 vs. BMI men 27.64 ± 5.89, *p* = 0.059).

The inclusion criteria for the study were met by patients who had not yet received ARNI therapy. The echocardiographic assessment included measurements of systolic and diastolic function parameters. Furthermore, laboratory diagnostics were performed to determine blood count, N-terminal pro-B-type Natriuretic Peptide (NT-proBNP), and lipid profile. In addition to the standard laboratory parameters, serum leptin levels were also measured. Follow-up assessments were performed after a median of 4 months [interquartile range (IQR): 3–7 months] following initiation of sacubitril/valsartan therapy.

### Serum leptin measurement

Venous blood was collected into serum tubes, allowed to clot for approximately 30 min, and centrifuged at 2,000 × g for 20 min at 4°C. The resulting serum was aliquoted and stored at −80°C until analysis. Serum leptin concentrations were determined using a commercially available enzyme-linked immunosorbent assay (ELISA; RD19100110, BioVendor—Laboratorni medicina a.s., Brno, Czech Republic) according to the manufacturer's instructions. Briefly, serum samples, standards, and quality controls were diluted 1:3. Absorbance was measured within 5 min at 450 nm using an iMark Microplate Absorbance Reader (Bio-Rad Laboratories, Vienna, Austria). Serum leptin concentrations were calculated from the standard curve using the optical density (OD) values of the serum samples.

### Ethics

Prior to inclusion in the study, all participants provided written informed consent. The study was conducted in accordance with the Declaration of Helsinki and received ethical approval from the local ethics committee (Salzburg: protocol code 415-E/2427/7-2019, approved June 2019; Münster: protocol code 2019-011-F-S, approved March 2019).

### Statistics

Statistical analyses were conducted using IBM SPSS Statistics version 29 (IBM Corp., Armonk, NY, USA). The normality of the distribution of continuous variables was tested using the Kolmogorov–Smirnov test. Baseline parameters that conformed to a normal distribution were presented as the mean ± standard deviation (SD). The means were then compared by means of a Student's t-test. Non-normally distributed baseline parameters were expressed as median and interquartile range (IQR) and were compared with the Mann–Whitney U-test. The dynamics of the parameters were assessed with dependent t-test for normally distributed variables or with Wilcoxon signed rank test for non-normally distributed variables. Absolute leptin change (*Δ*Leptin, ng/mL) was calculated by subtracting the baseline leptin concentration from the follow-up leptin concentration. Relative leptin change (%*Δ*Leptin) was calculated as the percentage change from baseline. Due to skewed distribution, NT-proBNP values were logarithmically transformed (log NT-proBNP) prior to analysis. To assess whether sex was independently associated with leptin dynamics under ARNI therapy, multivariable linear regression analyses were performed with *Δ*Leptin and %*Δ*Leptin as the dependent variables. Sex was included as the primary independent variable of interest. The models were adjusted for age, BMI, baseline left ventricular ejection fraction (LVEF), baseline log NT-proBNP and baseline serum creatinine. Categorical variables were expressed as numbers and percentages and compared by using the chi-squared test. The final stage of the study involved the assessment of the correlations between the variables of interest. This assessment employed both Pearson's correlation coefficient and Spearman's rank correlation coefficient. All *p*-values were two-sided, with statistical significance set at *p* < 0.05. An asterisk (*) indicates statistical significance (*p* < 0.05).

## Results

The baseline characteristics of the 20 women and 54 men (mean age: 59.1 ± 15.77 and 63.04 ± 11.31 years, respectively, *p* = 0.384) are shown in Table 1. Women more frequently had non-ischemic heart failure, whereas ischemic heart failure predominated in men *(p* = 0.009). The majority of patients were receiving adequate heart failure therapy at the time, consisting mainly of angiotensin-converting enzyme (ACE) inhibitors, angiotensin receptor blockers (ARBs), beta-blockers, mineral receptor antagonists, and diuretics. Patients not receiving beta-blocker and ACE inhibitor therapy had been recently diagnosed. None of the patients were receiving an ARNI, as was explicitly specified in the design of the study. The majority of patients were classified as NYHA stages II and III. Women and men were comparable with respect to NT-proBNP, LDL cholesterol, triglyceride levels and statin therapy. A significant difference was observed in leptin levels between female (39.78 ± 31.95 ng/mL) and male (10.39 ± 17.04 ng/mL) subjects (*p* < 0.001). However, the body mass index of the two groups was comparable. There was no significant difference between the two groups in regard to NYHA class. No significant association was observed between baseline leptin levels and baseline LVEF or NT-proBNP concentrations, for either sex.

**Table 1:** Baseline characteristics; median, lower and upper quartiles or standard deviation (Q1; Q3 or SD) and *n* (%).

Baseline Characteristics	Specification	Women, *N* = 20		Men, *N* = 54		*p*
		Results	Q1; Q3/SD or %	Results	Q1; Q3/SD or %	
Demographics	Age	59.1	(±15.77)	63.04	±11.31	0.384
Medical History	Ischemic Cardiomyopaty	5	25	32	59.3	0.009*
	Non-ischemic Cardiomyoapthy	15	75	22	40.7	0.009*
	Atrial fibrilation	6	30	14	25.9	0.635
	Dyslipidemia	11	55	31	57.4	0.971
	Current Smoking	5	25	21	38.9	0.265
	Diabetes	4	20	11	20.4	0.950
	Hypertension	13	65	27	50	0.165
	Family history of cardiovascular disease	4	20	13	24.1	0.789
Clinical Measurement	BMI	30.87	±6.84	27.64	±5.89	0.059
	NYHA class II	11	55	29	53.7	0.921
	NYHA class III	9	45	23	42.6	0.853
	NYHA class IV	0	0	2	3.7	0.383
	SBP (mmHg)	126.13	±22.2	124.45	±15.12	0.760
	Heart rate (bpm)	72.9	±20.72	71.20	±15.71	0.761
Treatment	Betablockers	18	90	47	83	0.729
	ACE inhibitors/ARBs	17	85	46	85.2	0.984
	Diuretics	13	65	29	53.7	0.384
	Aldosterone antagonists	13	65	38	70.4	0.658
	Statins	11	55	35	64.8	0.439
Laboratory	Leptin (ng/ml)	39.78	±31.95	10.39	±17.04	<0.001*
	NT-proBNP (pg/ml)	1,836.8	±2020.07	2,382.25	±2731.55	0.676
	Creatinine (mg/dl)	0.95	±0.28	1.13	±0.29	0.005*
	Total cholesterol (mg/dl)	164	±40.14	152.09	±42.49	0.195
	Triglyceride (mg/dL)	125.84	±61.80	132.44	±69.94	0.800
	HDL (mg/dL)	55.11	±20.43	50	±21.53	0.145
	LDL (mg/dl)	88.63	±31.39	83.09	±32.62	0.261

* *p* < 0.05. BMI, body mass index; NYHA class, New York Heart Association class; SBP, systolic blood pressure; ACE, angiotensin-converting enzyme iinhibitors; ARB, angiotensin II receptor blockers; NT-proBNP, N-terminal pro-B-type natriuretic peptide; HDL, high-density lipoprotein; LDL, low-density lipoprotein.

To account for potential baseline differences between men and women in the study cohort, multivariable linear regression analyses were performed adjusting for age, BMI, baseline LVEF, logNT-proBNP, and creatinine. None of the analyzed clinical variables showed a significant independent association with leptin dynamics. Sex was also not independently associated with absolute leptin change (B = −3.5 ng/mL, *p* = 0.58) or relative leptin change (B = + 48.3%, *p* = 0.44). These findings suggest that the observed leptin dynamics were not explained by differences in baseline clinical characteristics between the groups.

Echocardiographic measurements revealed that the left ventricular end-diastolic diameter (LVEDd) (55.90 ± 7.68 mm vs. 62.10 ± 8.81 mm, *p* = 0.003) and left atrial volume (35.46 ± 11.35 vs. 48.63 ± 20.68 ml/m^2^, *p* = 0.013) were significantly lower in women than in men. Tricuspid annular plane systolic excursion (TAPSE) was significantly higher in women (22.35 ± 5.26 vs. 18.10 ± 4.64, *p* = 0.007), though both were within the normal range. The remaining echocardiographic measurements exhibited no statistically significant differences (Table 2).

**Table 2:** Echocardiografic parameters in men and women at baseline.

Parameter	Women mean ± SD	Men mean ± SD	*p*
LVEF (%)	32.75 ± 8.25	29.13 ± 7.34	0.052
IVSd (mm)	9.50 ± 2.37	10.35 ± 2.7	0.326
LVEDd (mm)	55.90 ± 7.68	62.10 ± 8.81	0.003*
Left atrial volume (ml/m^2^)	35.46 ± 11.35	48.63 ± 20.68	0.013*
E/A	1.77 ± 1.28	1.49 ± 0.79	0.680
E/e‘	17.17 ± 9.73	12.78 ± 5.2	0.129
TAPSE (mm)	22.35 ± 5.26	18.10 ± 4.64	0.007*

* *p* < 0.05 (LVEF, left ventricular ejection fraction; IVSd, interventricular septum thickness at diastole; LVEDd, Left ventricular end-diastolic diameter; TAPSE, tricuspid annular plane systolic excursion).

### Dynamics of the parameters

The absolute and relative changes in the parameter after initiation of ARNI therapy are shown in Tables 3, 4.

**Table 3:** Parameters at baseline and follow-up for women and men.

Parameter	Women Baseline mean ± SD	Women Follow-up mean ± SD	*p*	Men Baseline mean ± SD	Men Follow-up mean ± SD	*p*
Leptin (ng/mL)	39.78 ± 31.95	50.10 ± 41.28	0.041*	10.39 ± 17.04	12.49 ± 16.62	0.006*
LVEF (%)	32.75 ± 8.25	41.13 ± 7.66	0.004*	29.13 ± 7.34	35.98 ± 10.68	< 0.001*
NT-proBNP (pg/ml)	1,836.80 ± 2,020.07	743.89 ± 687.91	0.015*	2,382.25 ± 2,731.55	1,641 ± 2,416.95	0.098
BMI (kg/m^2^)	30.87 ± 6.84	31.25 ± 9.76	0.213	27.64 ± 5.89	27.41 ± 5.55	0.264
NYHA class	2.50 ± 0.51	2.07 ± 0.64	0.414	2.51 ± 0.57	1.90 ± 0.64	<0.001*

* LVEF, left ventricular ejection fraction; NT-proBNP, N-terminal pro-B-type natriuretic peptide; BMI, body mass index; NYHA class, New York Heart Association class.

**Table 4:** Absolute and relative changes in biomarkers and clinical parameters after initiation of sacubitril/valsartan for women and men.

Parameter	Women *Δ* (mean ± SD or median)	Men *Δ* (mean ± SD or median)	*p*
Leptin (ng/mL)	11 (IQR 1.8;22.3)	3 (IQR 1;6.2)	0.131
Leptin (%)	+33.3% (IQR 2.32; 47.10)	+42.56% (IQR −4; 126.31)	0.430
LVEF (%)	9.18 ± 7.23	10.96 ± 6.83	0.376
NT-proBNP (pg/mL)	721 (IQR 271;1,504.5)	496 (IQR 164.75; 1,746.25)	0.792
NYHA class	0.615 ± 0.86	0.62 ± 0.83	0.745

*p* < 0.05. (LVEF, left ventricular ejection fraction; NT-proBNP, N-terminal pro-B-type natriuretic peptide; NYHA class, New York Heart Association class).

After a median follow-up of 4 months (IQR 3;7) following initiation of sacubitril/valsartan therapy, serum leptin concentrations increased significantly.

**Figure 1:**
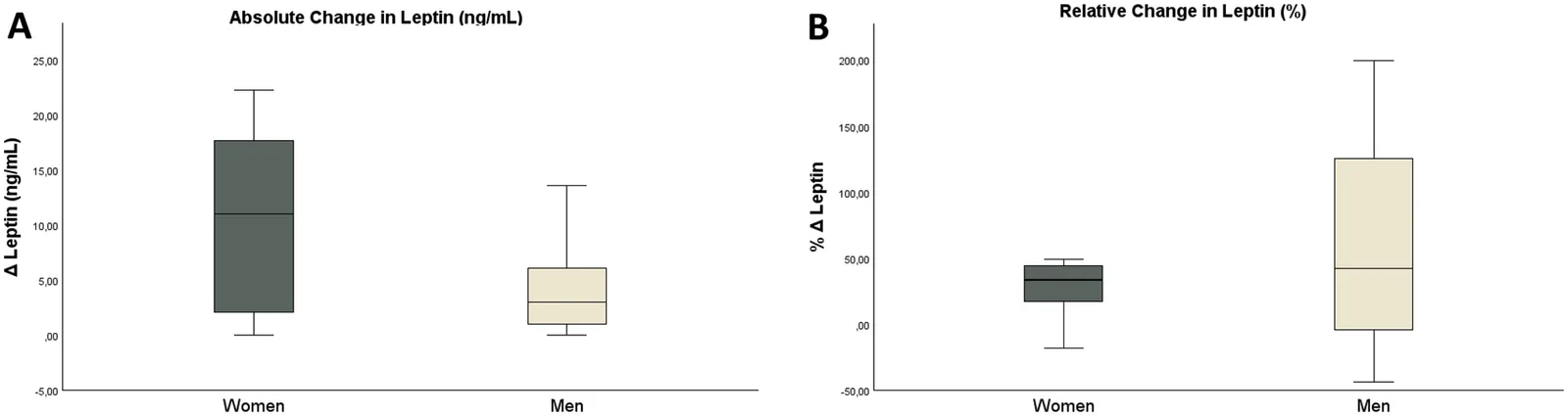
Absolute change and relative change in serum leptin concentration following initiation of sacubitril/valsartan therapy **(A)** absolute change in serum leptin concentration (ng/mL) in women and men. **(B)** Relative change in serum leptin concentration (% change from baseline) in women and men. Boxes represent the interquartile range with median values; whiskers indicate the data range.

In women leptin increased from 39.78 ± 31.95 to 50.10 ± 41.28 ng/mL (*p* = 0.041), whereas in men it increased from 10.39 ± 17.04 to 12.49 ± 16.62 ng/mL (*p* = 0.006) (Table 3). However, neither the absolute nor the relative change in leptin differed significantly between sexes (Table 4; Figure 1).

The absence of a substantial sex difference in relative leptin changes is likely explained by markedly higher baseline leptin concentrations in women, resulting in smaller relative percentage changes despite larger absolute increases. In addition, substantial interindividual variability was observed in men (Figures 1A,B).

Changes in leptin levels did not correlate significantly with changes in LVEF, NT-proBNP, or NYHA class. No significant differences in BMI were observed during follow-up in either sex, indicating that changes in leptin concentrations were independent of changes in body weight.

**Figure 2:**
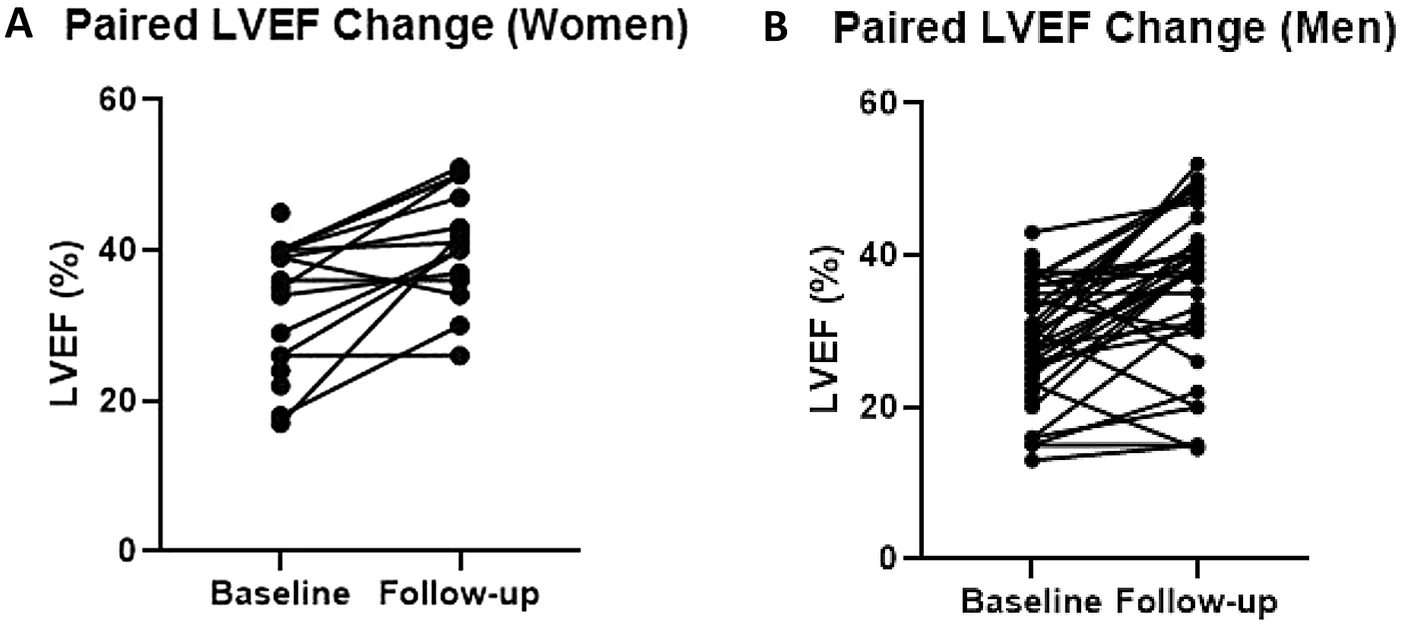
Paired dot plots illustrate the individual changes in LVEF (%) between baseline and follow-up for two sexes: **(A)** women (*p* = 0.004) and **(B)** men (*p* < 0.001). Each line corresponds to a single patient. (LVEF, left ventricular ejection fraction).

A significant improvement in left ventricular ejection fraction was observed in both woman and men subjects. At follow-up, LVEF remained numerically higher in women than in men; however, this difference did not reach statistical significance (41.13% ± 7.66% vs. 35.98% ± 10.68%, *p* = 0.105). Importantly, the magnitude of LVEF improvement (*Δ*LVEF) did not differ significantly between sexes (see Figure 2).

**Figure 3:**
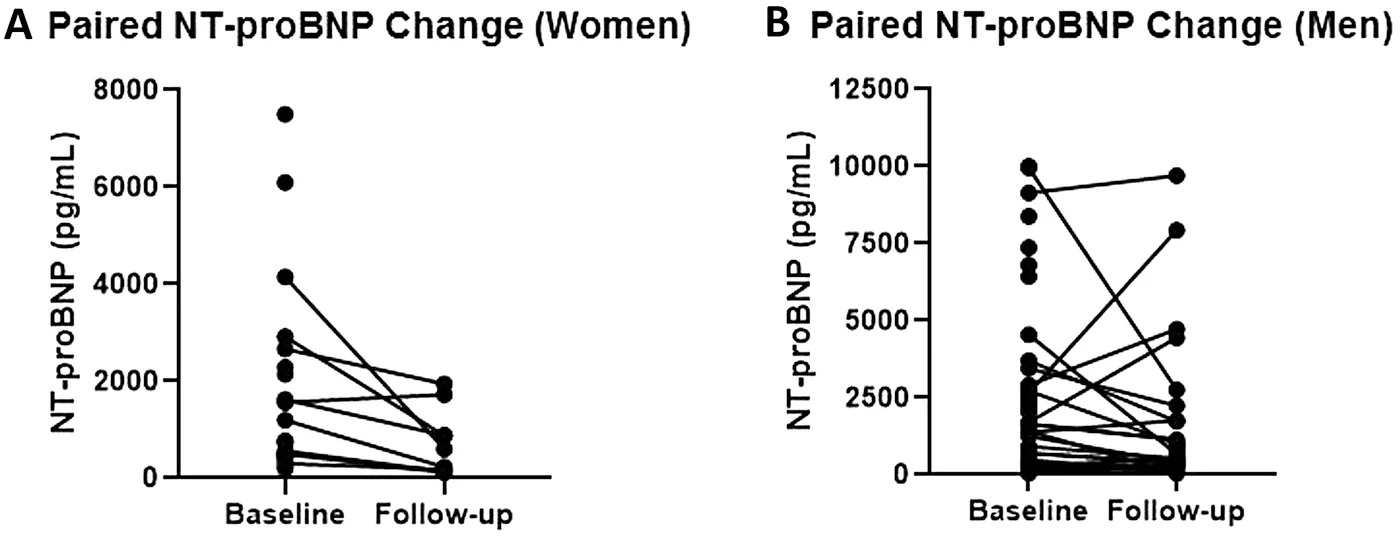
Paired dot plots illustrate individual changes in NT-proBNP between baseline and follow-up for two sexes: **(A)** women (*p* = 0.015) and **(B)** men (*p* = 0.098). Each line corresponds to a single patient. (NT-proBNP, N-terminal pro-B-type natriuretic peptide).

NT-proBNP concentrations demonstrated a statistically significant decrease in women (*p* = 0.015), while no such change was observed in men (*p* = 0.098). However, the magnitude of NT-proBNP change did not differ significantly between sexes (*p* = 0.792) (Figure 3).

**Figure 4:**
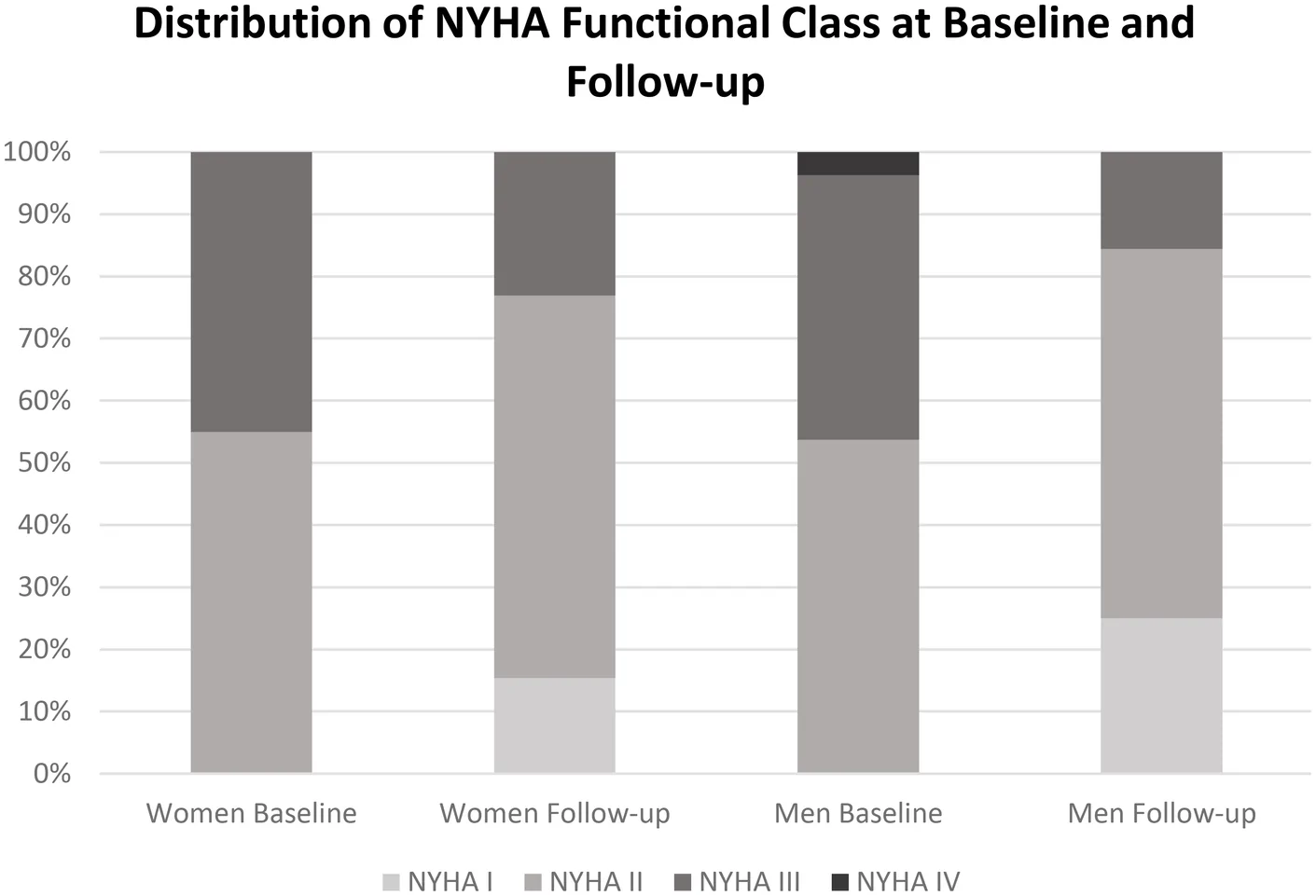
The stacked bar charts present the distribution of NYHA functional classes at baseline and follow-up, with the data presented separately for female and male subjects. (NYHA class, New York Heart Association class).

A significant improvement in NYHA functional class was observed in men, whereas the change in women did not reach statistical significance (*p* = 0.414). However, the magnitude of NYHA change did not differ significantly between sexes (*p* = 0.745) (Figure 4).

## Discussion

The aim of this study was to investigate whether there are gender-specific differences in leptin levels after initiation of sacubitril/valsartan therapy in patients with HFrEF. The study aimed to determine whether leptin responses to sacubitril/valsartan differ between men and women. A detailed analysis of leptin levels, NT-proBNP, and echocardiographic data after a short follow-up period were presented. Additionally, clinical data, including NYHA class, BMI, and other pertinent values, were evaluated.

Women diagnosed with HFrEF in our cohort presented with significantly elevated levels of leptin in comparison to male subjects, despite similar BMI values. This pattern is well established in the literature (26–29) and is generally attributed to greater leptin secretion in the female adipocytes. These sex-related differences in leptin concentrations are likely to be explained by hormonal regulation. Adipose tissue expresses androgen receptors (30), and androgens may influence leptin production. Several studies reported an inverse relationship between circulating testosterone levels and leptin receptor expression (28), and experimental data suggest that testosterone can inhibit leptin secretion (31). Moreover, testosterone-induced stimulation of lipolysis may be another mechanism through which androgens modulate leptin levels (30). Furthermore, oestrogen may enhance leptin production by promoting leptin gene expression and adipocyte differentiation (22, 32). These hormonal effects may partly explain the higher circulating leptin concentrations typically observed in women. Gender-specific factors that influence leptin production include the amount of adipose tissue and its distribution (33). The lack of a significant BMI difference between sexes in our cohort (with only a numerical trend toward higher BMI in women) suggests that the observed differences in leptin levels cannot be fully explained by differences in BMI alone and may instead reflect hormonal and cellular differences. A key finding of our study is the significant increase in leptin levels at a median of 4 (IQR: 3;7) months after the initiation of sacubitril/valsartan therapy. Although women had substantially higher leptin concentrations at baseline, the dynamics of leptin in response to ARNI therapy were comparable between the sexes, suggesting that sex influences baseline leptin levels, but not the response to therapy. The mechanisms underlying the effects of sacubitril are still not fully understood. As a neprilysin inhibitor, sacubitril blocks the enzyme responsible for degrading natriuretic peptides. This enhances the beneficial effects of these peptides in heart failure (34). Sacubitril has been shown to inhibit neprilysin, thereby blocking peptide degradation (34), a process that has been demonstrated to have a positive effect on heart failure. However, neprilysin is also expressed in adipocytes and correlates with obesity and insulin resistance (35) and appears to promote adipogenesis (36). By inhibiting neprilysin, sacubitril likely alters peptidase activity in adipose tissue, with downstream effects on adipose tissue function (36, 37).

The absolute increase in leptin levels was higher in women than in men, but this difference was not statistically significant. Similarly, the relative change in leptin concentrations did not differ significantly between the sexes. This finding parallels the pattern observed for absolute leptin levels, further indicating that sex determines baseline leptin concentrations without modifying the magnitude of the therapeutic response. Given the limited number of women in this cohort, however, this absence of a statistically significant sex difference should be interpreted with caution: it may reflect a true lack of sex-specific response, but it could equally reflect insufficient statistical power to detect a smaller, clinically relevant difference, particularly for the relative change estimate, which showed a wide interquartile range in men. Nonetheless, this pattern is consistent with previous studies showing that sex differences in leptin are primarily related to baseline adipocyte biology and hormonal regulation (26, 33).

The significance of leptin in heart failure remains to be fully understood. Several studies link elevated leptin to progression of heart failure or to higher cardiovascular risk (15, 38, 39), while others report higher mortality or poorer outcomes at elevated leptin levels (40). In the present cohort, leptin levels increased despite significant improvement in LV function, suggesting that elevated leptin concentrations do not necessarily reflect clinical deterioration. This observation is in line with the complex and still incompletely understood role of leptin in heart failure (39, 41).

The complex effects of leptin on energy metabolism, insulin sensitivity, and the sympathetic nervous system (33, 42–45) suggest that the observed increase during sacubitril/valsartan treatment could indicate either metabolic adaptation or sympathetic activation. However, persistent sympathetic activation could lead to adverse cardiometabolic outcomes over time (42, 43). In the present study, leptin dynamics were not independently associated with baseline clinical characteristics, suggesting that the observed increase in leptin was not explained by differences in disease severity, body mass, or renal function between the groups. Nevertheless, it remains unclear whether the observed increase in leptin indicates beneficial metabolic adaptation or detrimental neurohormonal activation.

Changes in NT-proBNP and NYHA class showed different patterns within each sex. While the magnitude of change did not differ significantly between women and men, the two parameters showed distinct patterns within each sex: NT-proBNP decreased significantly in women but not in men, while NYHA class improved significantly only in men — though in neither case did the between-sex difference in magnitude reach significance. Differences in NT-proBNP dynamics between sexes have been described in previous studies (46). Women have a higher prevalence of non-ischemic heart failure and frequently demonstrate lower NT-proBNP levels. Pharmacokinetic differences may also play a role, since women can process and eliminate medications differently. Indeed, ARNI therapy has been shown to have sex-specific effects on biomarkers and remodelling (47–49). Earlier improvement in LV function can lead to a decrease in NT-proBNP (49). Higher neprilysin activity in women has also been suggested (50), which could further modify the pharmacodynamics of sacubitril/valsartan. The significant decrease in NT-proBNP levels in women could reflect any combination of etiology, an accelerated remodeling response, or pharmacokinetic differences.

NYHA class improved significantly in men but not in women, although again, the absolute change did not differ significantly between sexes. The NYHA classification is subjective, and symptom perception may differ between men and women. Previous studies have shown that psychosocial factors partially explain gender differences in health-related quality of life among patients with heart failure, which may also influence patient-reported symptom burden (51). The significant NYHA improvement observed in the male population may be explained by subjective differences in perception and different comorbidity profiles.

We found sex-related differences in the pattern of biomarker and symptom changes during sacubitril/valsartan therapy, with female subjects demonstrating a greater decrease in NT-proBNP and male subjects demonstrating a greater improvement in NYHA class. These results align with those of other studies; however, they do not provide sufficient evidence to conclude that sacubitril/valsartan is more effective in women than in men. The need for additional prospective and randomized studies in this area is evident.

Women in our study had significantly higher levels of circulating leptin than men at both baseline and follow-up. Leptin increased significantly under sacubitril/valsartan in both sexes and this increase occurred independently of sex. However, there were no significant differences in absolute or relative leptin changes between women and men, suggesting that the leptin response to ARNI therapy is sex-independent.

## Limitations

This study has several limitations. The study population was relatively small and the study was observational in design. In addition, the unequal distribution of men and women in the study cohort may have reduced the statistical power to detect potential sex-specific differences. The study was conducted during the period of the coronavirus pandemic, which resulted in a limited number of patients being enrolled. Follow-up appointments were scheduled according to the patient's clinical state and the manifestation of symptoms. Consequently, a number of follow-up visits were postponed due to the pandemic.

The hormonal profiles of the women, including menopausal status and hormone replacement therapy, were not documented. Therefore, the potential influence of sex hormones on baseline leptin concentrations and leptin dynamics could not be assessed, which may have affected the interpretation of the observed sex differences. The aetiology of heart failure in the study population was heterogeneous. Although adjustments for several clinical variables were performed, residual confounding cannot be excluded.

Furthermore, the study population was recruited from two Central European centres and consisted predominantly of patients of Caucasian origin. Therefore, the generalizability of our findings to other ethnic populations may be limited.

In addition, body composition beyond BMI was not assessed in detail either, limiting the ability to fully interpret the metabolic determinants of leptin levels.

Finally, this study had no control group. Due to contemporary guideline recommendations at the time of the study, most eligible patients were switched to sacubitril/valsartan as soon as possible, making the formation of a control group difficult.

## Conclusion

This observational analysis revealed that women had significantly higher circulating leptin levels than men. The initiation of ARNI therapy was associated with a significant increase in leptin concentrations in both sexes. However, there was no significant difference in the magnitude of leptin change between women and men, suggesting that, although sex influences baseline leptin levels, the leptin response to ARNI therapy appears to be independent of sex.

Although improvements in NT-proBNP and NYHA class showed different patterns within each sex, the magnitude of these changes did not differ significantly between women and men.

Further prospective studies involving larger patient cohorts are required to clarify the clinical significance of leptin changes in patients with HFrEF who are treated with ARNI therapy.

## Data Availability

The raw data supporting the conclusions of this article are available from the corresponding author upon reasonable request.
